# Randomized Efficacy Trial of Conventional, TCM Herb, and TEAS on Bone Marrow Suppression in Patients with Small Cell Lung Cancer after Initial Chemotherapy

**DOI:** 10.1155/2021/2693472

**Published:** 2021-02-02

**Authors:** Fangchao Zhao, Zengying Wang, Yanlin Gao, Yusi Wu, Jianming Liu, Shuangliang He

**Affiliations:** ^1^Department of Thoracic Surgery, Tangshan People's Hospital, North China University of Science and Technology, Tangshan 063000, China; ^2^The Graduate School of Hebei Medical University, Shijiazhuang 050000, China; ^3^Department of Urology, Second Hospital of Hebei Medical University, Shijiazhuang 050000, China; ^4^Department of Nursing, Tangshan People's Hospital, North China University of Science and Technology, Tangshan 063000, China; ^5^Department of Anesthesiology, Tangshan People's Hospital, North China University of Science and Technology, Tangshan 063000, China

## Abstract

**Objective:**

To compare the efficiency of transcutaneous electrical acupoint stimulation (TEAS) with those of conventional and TCM herb on bone marrow suppression in small cell lung cancer (SCLC) patients after initial chemotherapy.

**Methods:**

We recruited 139 participants with pathologically confirmed SCLC who had not received chemotherapy. The conventional group (*n* = 37) received gemcitabine and cisplatin chemotherapy and routine care. The TCM herb group (*n* = 35) received 3 Diyushengbai tablets thrice a day for one day prior to chemotherapy and maintained during the trial. The TEAS group (*n* = 42) received TEAS at a frequency of 65–100 Hz with a pulse width of 100–200 *μ*sec. Acupoints were selected from Dazhui (DU14), Geshu (BL17), Zusanli (ST36), Sanyinjiao (SP6), and Hegu (LI4) and were treated on days 1, 2, 3, 5, 8, 14, 21, and 28 of chemotherapy for 30 min each day. All three groups underwent a 28-day treatment for a total of one treatment course. Changes in the white blood cell, neutrophil, platelet, and hemoglobin indices on day 1 before chemotherapy and days 5, 8, 11, 14, 21, and 28 days after chemotherapy were compared among the groups. Comfort levels of patients on day 1 before chemotherapy and days 5, 11, and 21 after chemotherapy were observed.

**Results:**

Compared with the conventional group, the white blood cell counts in the TEAS group on days 8 (7.07 ± 2.11 vs. 5.97 ± 2.10 × 10^9^/L) and 14 (6.14 ± 1.51 vs. 5.07 ± 2.41 × 10^9^/L) of chemotherapy and that in the TCM herb group on day 14 (6.63 ± 3.44 vs. 5.07 ± 2.41 × 10^9^/L) of chemotherapy were increased (*P* < 0.05). Compared with the conventional group, the neutrophil count in the TEAS group on days 5 (4.28 ± 1.54 vs. 3.01 ± 1.41 × 10^9^/L), 8 (3.75 ± 1.21 vs. 2.77 ± 1.17 × 10^9^/L), 11 (3.46 ± 1.31 vs. 2.31 ± 1.24 × 10^9^/L), 14 (3.18 ± 1.29 vs. 2.07 ± 1.14 × 10^9^/L), and 21 (4.67 ± 1.31 vs. 3.58 ± 1.23 × 10^9^/L) of chemotherapy and that in the TCM herb group on day 5 (3.88 ± 1.05 vs. 3.01 ± 1.41 × 10^9^/L) of chemotherapy were increased (*P* < 0.05). Compared with the conventional group, the platelet count of patients in the TEAS group increased on days 5 (264.7 ± 64.1 vs. 201.0 ± 55.7 × 10^9^/L), 8 (251.3 ± 74.9 vs. 188.2 ± 65.8 × 10^9^/L), 11 (236.7 ± 74.9 vs. 181.3 ± 84.3 × 10^9^/L), and 14 (238.3 ± 75.9 vs. 192.8 ± 95.8 × 10^9^/L) of chemotherapy (*P* < 0.05). Compared with the TCM herb group, the platelet count in the TEAS group increased on days 5 (264.7 ± 64.1 vs. 216.3 ± 57.9 × 10^9^/L), 8 (251.3 ± 74.9 vs. 213.7 ± 70.3 × 10^9^/L), 11 (236.7 ± 74.9 vs. 181.3 ± 84.3 × 10^9^/L), and 21 (254.8 ± 81.8 vs. 213.9 ± 82.6 × 10^9^/L) of chemotherapy (*P* < 0.05). Compared with the conventional group, the hemoglobin level in the TCM herb group increased on day 14 (135.03 ± 28.06 vs. 122.09 ± 12.63 g/L) of chemotherapy (*P* < 0.05). Compared with the conventional group, the comfort score of the TEAS group increased on days 5 (78.31 ± 10.21 vs. 70.18 ± 9.34 score) and 11 (80.07 ± 10.44 vs. 72.11 ± 9.47 score) of chemotherapy (*P* < 0.05).

**Conclusion:**

TEAS is an effective and safe treatment modality for improving bone marrow suppression in SCLC patients after initial chemotherapy. TEAS improved comfort levels more effectively than did conventional and TCM herb.

## 1. Introduction

Lung cancer is the most common malignant tumor in China and worldwide, with its incidence and mortality increasing in recent years [1, 2]. Chemotherapy is currently the main treatment for small cell lung cancer (SCLC), of which platinum-based double-agent chemotherapy could significantly reduce mortality in patients with SCLC [3]. However, >90% of chemotherapeutic drugs can cause bone marrow suppression, which is manifested by a decrease in hemoglobin, white blood cells, and platelets. Chemotherapy-induced bone marrow suppression is the most common side effect in the clinical treatment of tumors. It not only significantly affects the clinical symptoms and immune status for patients with SCLC but also may result in dose decrease or even the treatment discontinuation and then worsen the disease progression [4].

Diyushengbai tablet, a traditional Chinese herbal medicine, which was widely used for cooling blood and hemostasis, is now being used clinically for more than 10 years for the treatment of radiotherapy- and chemotherapy-induced leukopenia. Studies have shown that the Diyushengbai tablet increases levels of white blood cells (WBCs) and platelets and relieves bone marrow depression in leukopenic mice [5, 6].

Alternative therapies are also one of the most potential modalities for cancer patients to treat chemotherapy-related side effects. These interventions include acupuncture, acupoint injection, electroacupuncture, and transcutaneous electrical nerve stimulation (TENS) [7]. Liu et al.[8] showed that Zusanli (ST36) acupoint injections with dexamethasone combined with oral tablets significantly increased white blood cell and neutrophil counts. Lin et al.[9] demonstrated that Sanyinjiao (SP6) acupoint injections enhance the effects of cyclophosphamide by inhibiting tumor growth and relieving bone marrow depression and any adverse effects of the drug on the immune system. Acupuncture and acupoint injections at ST36, SP6, Xuehai, Qihai, and Guan Yuan have also been reported to improve chemotherapy-induced bone marrow suppression [10]. However, the abovementioned interventions are invasive, with patients feeling pain and thus having poor compliance with treatment.

TEAS is an improved type of therapy that combines acupuncture points and TENS. The effect of TEAS is similar to acupuncture, although the deepening of its treatment theory and research progress was done on the basis of acupuncture, with this method being noninvasive and having a higher clinical acceptance [11]. However, no data on the effectiveness of TEAS for treating chemotherapy-induced bone marrow suppression in patients with SCLC are available.

This prospective study explored the use of TEAS as an alternative treatment option for chemotherapy-induced bone marrow suppression in SCLC patients after initial chemotherapy. We compared the efficiency of this treatment method with those of conventional and TCM herb.

## 2. Methods

This is a 3-site, prospective, randomized, controlled trial study conducted in the Tangshan people's Hospital Affiliated to North China University of Science and Technology from July 2019 to August 2019. The study followed the declaration of Helsinki and was approved by the Medicine Research Ethics Committee of the Tangshan people's Hospital Affiliated to North China University of Science and Technology (No: RMYY-LLKS-2019-023). The information of the study was explained to all enrolled participants, and written informed consent was obtained from each participant.

### 2.1. Inclusion Criteria

This study recruited patients with SCLC who received chemotherapy for the first time. Inclusion criteria were as follows: (1) extensive-stage small cell lung cancer who had not received chemotherapy and were diagnosed pathologically; (2) Karnofsky Performance scores (KPSs) ≥70 points; (3) no severe cardiovascular, cerebrovascular, or hematological diseases; and (4) good cognitive function and language ability. Exclusion criteria were as follows: (1) skin reddening or fester at the acupoints of Dazhui (DU14), Geshu (BL17), ST36, SP6, and Hegu (LI4); (2) surgery performed in the abovementioned acupoints; (3) a history of hypertension, diabetes mellitus, epilepsy, currently pregnant, lactating, or fertile, or participating in other clinical trials; and (4) bone marrow suppression before chemotherapy.

### 2.2. Randomization

Patients were assigned to the experimental groups using a table of computer-generated random numbers. According to the order of admission, the patients were randomly allocated to each of the three groups: the conventional group, the TCM herb group, and the TEAS group.

### 2.3. Treatment Methods

All patients received etoposide and cisplatin combination chemotherapy (etoposide 100 mg/m^2^, intravenous [i.v.] drip, day 1 to day 3; cisplatin 30 mg/m^2^, i.v. drip, day 1 to day 3) in addition to hydration therapy. Each of the three groups received routine therapy with omeprazole injection (40 mg, i.v. drip, one a day) during chemotherapy. Four milligrams of tropisetron hydrochloride with 100 mL of sodium chloride and 3 mg of dexamethasone were administered intravenously 30 min before chemotherapy.

The following aspects were taken into consideration while the conventional group received routine nursing care: (1) the condition of the patients needs to be observed closely and the possible complications should be prevented and the doctors should be informed of these complications as early as possible; (2) the patients were encouraged to take food rich in protein and vitamins; (3) the patients should be routinely administered with antiemetics (e.g., ondansetron) and gastroprotective agents (e.g., omeprazole).

For the TCM herb group, in addition to routine nursing care, patients took a Diyushengbai tablet (Aobang Pharmaceutical Co, LTD, Sichuan, China; batch number of 20180413; t.i.d., three tablets per time) before chemotherapy.

For the TEAS group, in addition to routine nursing care, acupoint positioning was carried out by TCM clinical experts. TEAS was applied to five pairs of acupoints, including DU14, BL17, ST36, SP6, and LI4. TEAS was performed on days 1, 2, 3, 5, 8, 14, 21, and 28, using the WOND Multifunctional Nerve Therapy (Huayi Medical Instrument Co., Ltd, Shanghai, China) with the following parameters: pulse width of 0.2 ms, frequency of 100 Hz, stimulation duration of 10 s, stimulation interval of 3 seconds, stimulation quantity of 20 to 25 mA, and maximum feedback stimulation quantity of 30 mA. Patients were in the supine position, and their acupoint positions were wiped with 95% ethanol for partial removal of grease. Subsequently, electrode patches with a diameter of 3 cm were applied to five pairs of acupoints. The current intensity was adjusted to maintain visible muscle twitches in the locations of the acupoints although this was not beyond the patients' maximum tolerance. The current intensity was increased according to the patients' decision or on the degree of their muscle twitches. Generally, each therapy session lasted for 30 min.

Each participant filled in an electrical acupoint stimulation record card, which listed the current date, treatment frequency and intensity, and any complications from the session.

### 2.4. Measurements

Routine blood indices, such as white blood cell, neutrophil, platelet, and hemoglobin levels, were the primary observation indicators. An ADVIA120 automatic hematology analyzer (Bayer, Leverkusen, Germany) was used for routine blood examination of indicators of bone marrow suppression, including white blood cell (×10^9^/L), neutrophil (×10^9^/L), and platelet counts (×10^9^/L), and hemoglobin (g/L) levels. Tests were performed on the day before the start of chemotherapy and on days 5, 8, 11, 14, 21, and 28 of the study period.

The secondary observation indicator was the degree of comfort. The degree of comfort was assessed on the day before chemotherapy and on days 5, 11, and 21 of the experimental period. The Comfort Scale (questionnaire GCQ, general comfort), which was designed by the American comfort nursing expert, Kolcaba, has good reliability (Cronbach's alpha = 0.96). The content validity index was 0.86. There are 30 items in this scale, including 5 physical dimensions, 10 mental dimensions, 8 social-cultural dimensions, and 7 environmental dimensions. Each of the items is classified using a 1–4 Likert scale. The lowest score is 30 points, and the highest score is 120 points. The higher the score is, the more comfort the patient is experiencing. A total score of ≤60 was considered a low comfort level, a score between 60 and 90 points was considered a moderate comfort level, and a score of ≥90 points was considered a high comfort level [12]. A preliminary experiment was conducted in 30 patients with NSCLC before the first visit, and the reliability coefficient of the scale was calculated; Cronbach's alpha = 0.924 indicated high internal consistency.

### 2.5. Statistical Analysis

Statistical analyses were performed using SPSS 22.0. Enumeration data are presented as absolute quantities. Measurement data are presented as means ± standard deviations (SDs). A one-way analysis of variance was used to compare the patients' according to their ages (means ± SDs), course of the disease, body mass indices, white blood cell, neutrophil count, platelet, and hemoglobin levels, and comfort scores among the three groups. Three independent samples' nonparametric tests were used to analyze the KPS score, type, and stage. Statistical significance was defined as a *P* value <0.05.

## 3. Results

### 3.1. Patients' Baseline Characteristics

**Figure 1:**
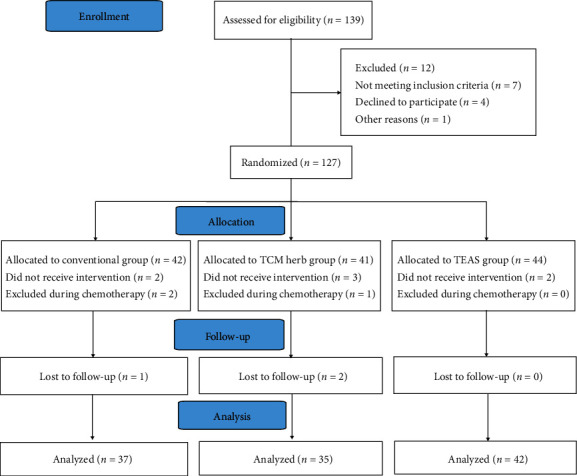
Flow chart of the study. The flowchart shows the study procedures and the number of patients. TEAS, transcutaneous electrical acupoint stimulation.

A total of 139 patients were screened in this study as shown in Figure 1. Of these, 12 patients (8.6%) dropped out of the study, 7 patients did not meet the inclusion criteria, 1 patient has other reasons, and 4 patients declined to receive TEAS. Finally, 127 patients participated in the study, 48 of whom were randomized to the conventional group, 41 to the TCM herb group, and 44 to the TEAS group. 13 patients discontinued the study after randomization, 2 of whom were in the TEAS group, 6 in the TCM herb group, and 5 in the conventional group. 2 patients did not receive TEAS because they were not able to tolerate the sensation of “De-Qi”. 3 patients refused to take Diyushengbai tablets during chemotherapy. 2 patients did not receive conventional treatment. 3 patients were excluded during chemotherapy because of bone marrow suppression, which required more treatment. 3 patients were lost to follow-up and we did not obtain the comfort scores. At last, 114 patients completed the study and their records were analyzed. The three groups were comparable according to demographic characteristics and surgical information (*P* > 0.05) (Table 1).

### 3.2. Changes in White Blood Cell Counts

There was no significant difference in white blood cell counts among the three groups (*P* > 0.05) on the day before chemotherapy. Compared with before chemotherapy, the white blood cell counts of the patients in the three groups decreased at each time point after chemotherapy (*P* < 0.05). Compared with the conventional group, the white blood cell counts in the TEAS group on days 8 (7.07 ± 2.11 vs. 5.97 ± 2.10 × 10^9^/L) and 14 (6.14 ± 1.51 vs. 5.07 ± 2.41 × 10^9^/L) of chemotherapy and those in the TCM herb group on day 14 (6.63 ± 3.44 vs. 5.07 ± 2.41 × 10^9^/L) of chemotherapy were increased (*P* < 0.05). There was no significant difference in white blood cell counts between the TEAS and TCM herb groups at various time points after chemotherapy (*P* > 0.05) (Table 2).

### 3.3. Changes in Neutrophil Counts

There was no significant difference in neutrophil counts among the three groups on the day before chemotherapy (*P* > 0.05). Compared with before chemotherapy, the neutrophil counts of the patients in each group decreased at each time point after chemotherapy (*P* < 0.05). Compared with the conventional group, the neutrophil count in the TEAS group on days 5 (4.28 ± 1.54 vs. 3.01 ± 1.41 × 10^9^/L), 8 (3.75 ± 1.21 vs. 2.77 ± 1.17 × 10^9^/L), 11 (3.46 ± 1.31 vs. 2.31 ± 1.24 × 10^9^/L), 14 (3.18 ± 1.29 vs. 2.07 ± 1.14 × 10^9^/L), and 21 (4.67 ± 1.31 vs. 3.58 ± 1.23 × 10^9^/L) of chemotherapy and that in the TCM herb group on day 5 (3.88 ± 1.05 vs. 3.01 ± 1.41 × 10^9^/L) of chemotherapy were increased (*P* < 0.05). There was no significant difference in neutrophil counts between the TEAS and TCM herb groups at various time points after chemotherapy (*P* > 0.05) (Table 3).

### 3.4. Changes in Platelet Counts

There was no significant difference in platelet counts among the three groups (*P* > 0.05) on the day before chemotherapy. Compared with before chemotherapy, the platelet count in the TCM herb group decreased at all time points after chemotherapy (*P* < 0.05), the platelet count in the routine group decreased on days 5, 8, 11, and 14 after chemotherapy (*P* < 0.05), and there was no significant difference in platelet counts in the TEAS group at all time points after chemotherapy (*P* > 0.05). Compared with the conventional group, the platelet count of patients in the TEAS group increased on days 5 (264.7 ± 64.1 vs. 201.0 ± 55.7 × 10^9^/L), 8 (251.3 ± 74.9 vs. 188.2 ± 65.8 × 10^9^/L), 11 (236.7 ± 74.9 vs. 181.3 ± 84.3 × 10^9^/L), and 14 (238.3 ± 75.9 vs. 192.8 ± 95.8 × 10^9^/L) of chemotherapy (*P* < 0.05), and there was no significant difference between the TCM herb and conventional groups at all time points (*P* > 0.05). Compared with the TCM herb group, the platelet count in the TEAS group increased on days 5 (264.7 ± 64.1 vs. 216.3 ± 57.9 × 10^9^/L), 8 (251.3 ± 74.9 vs. 213.7 ± 70.3 × 10^9^/L), 11 (236.7 ± 74.9 vs. 181.3 ± 84.3 × 10^9^/L), and 21 (254.8 ± 81.8 vs. 213.9 ± 82.6 × 10^9^/L) of chemotherapy (*P* < 0.05) (Table 4).

### 3.5. Changes in Hemoglobin Concentrations

There was no significant difference in hemoglobin levels among the three groups on the day before chemotherapy (*P* > 0.05). Compared with before chemotherapy, there was no significant difference in hemoglobin levels among the three groups after chemotherapy (*P* > 0.05). Compared with the conventional group, the hemoglobin level in the TCM herb group increased on day 14 (135.03 ± 28.06 vs. 122.09 ± 12.63 g/L) of chemotherapy (*P* < 0.05), and there was no significant difference between the TEAS and conventional groups (*P* > 0.05) (Table 5).

### 3.6. Comfort Score

There was no significant difference in comfort scores among the three groups (*P* > 0.05) on the day before chemotherapy. Compared with before chemotherapy, the comfort score of the conventional group decreased on days 5 and 11 of chemotherapy (*P* < 0.05), and the comfort score of the TEAS group at each time point after chemotherapy was not significantly different from that before chemotherapy (*P* > 0.05). Compared with the conventional group, the comfort score of the TEAS group increased on days 5 (78.31 ± 10.21 vs. 70.18 ± 9.34 score) and 11 (80.07 ± 10.44 vs. 72.11 ± 9.47 score) of chemotherapy (*P* < 0.05), and there was no significant difference between the TCM herb and conventional groups at all time points (*P* > 0.05). There was no significant difference in comfort score between the TEAS and TCM herb groups at various time points after chemotherapy (*P* > 0.05) (Table 6).

## 4. Discussion

This study showed that TEAS can prevent chemotherapy-induced bone marrow suppression in patients with SCLC. The symptoms of bone marrow depression include physical decline, fatigue, weakness, sweating, dizziness, tinnitus, palpitation, severe palpitation, anorexia, nausea, vomiting, and weight loss [13]. These symptoms belong to the TCM asthenia category, which includes specific symptoms such as spleen and kidney weakness, Qi, and blood deficiency [14]. Yu et al.[15] showed that bone marrow suppression could lead to depressed emotion, thus affecting patients' quality of life and chemotherapy compliance. Severe bone marrow suppression may cause termination or failure of treatment. Therefore, effective prevention of chemotherapy-induced bone marrow suppression is of great importance to improve patients' quality of life, strengthen their confidence in overcoming the disease, and ensure successful chemotherapy.

At present, TCM mainly uses point injection therapy to treat bone marrow suppression. The theory of TCM considers chemotherapy as a process able to overcome disease and protecting the viscera, bone marrow, blood, Yin, and Yang, although it often results in impairment of spleen, stomach, liver, and kidney functions. Fu et al.[16] showed that SP6 acupoint injection enhances the effects of cyclophosphamide by inhibiting tumor growth and relieving bone marrow suppression and the drug's adverse effects on the immune system. Acupuncture and acupoint injections at ST36, SP6, Xuehai, Qihai, and Guanyuan have also been reported to improve chemotherapy-induced bone marrow depression [17]. However, the abovementioned interventions are invasive, with patients reportedly experiencing pain which leads to poor compliance with treatment. Thus, the clinical efficacies of these interventions need further study. TEAS combines and fully utilizes the advantages of both TENS and the meridian acupoint theory. Its main functions are to excite neuromuscular tissue, repair the nerve conduction system, and promote absorption of inflammatory substances, among many other functions. This study used TEAS in patients with SCLC receiving chemotherapy and showed that low-frequency electrical acupoint stimulation can prevent chemotherapy-related bone marrow suppression and improve the comfort of patients.

TEAS significantly increased white blood cell and neutrophil counts. The DU14 acupoint is located in the seventh cervical spinous process which regulates Qi and blood and improves white blood cell counts with acupoint LI4 by 80–90% [18]. Electrical stimulation at ST36 induces corticotropin-releasing factors, which enhance the functions of the pituitary gland, adrenal cortex, the sympathetic–adrenal system, and their secretion of vasoactive substances, ultimately adjusting the pressure in the marrow cavity, regulating bone marrow blood flow, and promoting the formation of white blood cells and neutrophils [19]. White blood cell and neutrophil counts decreased from day 1 of chemotherapy and reached their lowest levels on day 14. On day 21, white blood cell and neutrophil counts began to increase. Compared with the conventional group, white blood cell count in the TEAS group increased on days 8 and 14 of chemotherapy. Neutrophil count in the TEAS group increased on days 5, 8, 11, 14, and 21 of chemotherapy. This indicates that TEAS can increase both white blood cell and neutrophil counts.

Some studies [20] suggested that simultaneous stimulation at BL17, SP6, and LI4 can be used for the treatment of primary thrombocytopenic purpura, which could significantly improve the clinical symptoms of patients and promote recovery of platelet counts. Electrical acupoint stimulation at SP6 can nourish the Yin and blood. BL17 (located at the seventh thoracic spinous process, 1.5 inches from the median line o) is the rally point of the eight points of the blood. Stimulating this acupoint nourishes the blood and Qi. Studies have shown that acupuncture at LI4 increases the platelet count. Stimulation at LI4 and DU14 significantly reduces leukocyte count. On days 5, 8, and 11 of this study, platelet counts were significantly higher in the TEAS group than those in the conventional and TCM herb groups. On day 21, platelet counts were significantly higher in the conventional and TEAS groups than those in the medication group, indicating that TEAS may help prevent thrombocytopenia after chemotherapy. Hou et al. [21] found that electrical acupoint stimulation could increase the production of red blood cells and hemoglobin. However, this study found that low-frequency electrical acupoint stimulation had little influence on red blood cells and hemoglobin, which may be because of the study's small sample size or short follow-up time (≤120 days).

Additionally, the comfort level of the patient during chemotherapy is an important indicator affecting treatment compliance [22, 23]. The meridian theory of TCM [24] shows that stimulation of the relevant acupuncture points of the meridian system can balance Yin and Yang, support health, and resist evil, thus achieving efficacy in promoting blood circulation to remove blood stasis and eliminate inflammation. TEAS can improve microcirculation, promote absorption of inflammatory substances, and improve cell oxygenation and metabolism. It can also promote the recovery of intestinal peristalsis, reduce abdominal distension, and promote defecation [25]. Stimulation at ST36 and SP6 can treat vomiting, abdominal distension, constipation, stomachache, lower limb arthralgia, and insomnia. Comfort scores on days 5 and 11 of chemotherapy in the TEAS group were higher than those in the conventional group, confirming that TEAS can significantly improve the comfort of patients with SCLC after chemotherapy.

This study has 2 limitations. First, blood counts only went down a small amount and no neutropenia or other concerning levels were reported, a potential benefit that would need further studies. Second, this study only evaluated the short-term efficacy and comfort of TEAS for myelosuppression in SCLC patients undergoing initial chemotherapy, because it did not consist of follow-up evaluation for all chemotherapy cycles. A more important study would be to assess the intervention over time to see if neutropenia, anemia, and thrombocytopenia were prevented after 4–6 cycles of treatment.

## 5. Conclusion

TEAS is an effective and safe treatment modality for improving bone marrow suppression in SCLC patients after initial chemotherapy. TEAS improved comfort levels more effectively than did conventional and TCM herb.

## Figures and Tables

**Table 1:** Demographic data and cancer details.

Index	Conventional (*n* = 37)	TCM herb (*n* = 35)	TEAS (*n* = 42)	*χ* ^*2*^	*P* value
Gender				0.536	0.464
Male	25	21	26
Female	12	14	16
Age (years)	57.31 ± 9.72	58.64 ± 8.29	58.19 ± 9.32	0.496	0.481
Smoking history				0.209	0.748
No	15	11	15
Yes	22	24	27
BMI (kg/m^2^)	24.34 ± 6.41	25.31 ± 6.29	24.05 ± 7.12	0.147	0.702
KPS				0.053	0.818
<80	3	4	9
≥80	34	31	33
Clinical type				0.640	0.641
Adenocarcinoma	19	18	25
Squamous cell carcinoma	16	16	15
Other	2	1	2
Degree of differentiation				0.216	0.973
Highly differentiated	3	4	8
Moderately differentiated	24	26	25
Poorly differentiated	10	5	9
TNM stage				0.556	0.634
I–III	33	33	37
IV	4	2	5		

Values are mean (SD) or number. A one-way analysis of variance was used to compare the patients' according to their ages (means ± SDs), course of disease, and body mass indices among the three groups. Three independent samples' nonparametric tests were used to analyze the KPS score, type, and stage. BMI, body mass index (calculated as weight in kilograms divided by height in meters squared); KPS, Karnofsky performance score; TNM, tumor, node, and metastasis.

**Table 2:** Comparison of the changes in white blood cell count among the three groups (×10^9^/L, $\bar{x}$ *±* *s*).

Groups	1 d before	Day 5	Day 8	Day 11	Day 14	Day 21	Day 28
Conventional (*n* = 37)	7.37 ± 2.54	6.52 ± 2.6^a^	5.97 ± 2.10^a^	5.51 ± 2.34^a^	5.07 ± 2.41^a^	6.18 ± 2.23^a^	6.46 ± 2.12^a^
TCM herb (*n* = 35)	7.56 ± 2.91	6.35 ± 2.46^a^	6.48 ± 2.58^a^	5.73 ± 2.21^a^	6.63 ± 3.44^a,b^	6.10 ± 2.47^a^	5.97 ± 2.74^a^
TEAS (*n* = 42)	7.32 ± 2.63	6.57 ± 1.91^a^	7.07 ± 2.11^a,b^	6.32 ± 2.41^a^	6.14 ± 1.51^a,b^	6.52 ± 1.74^a^	6.45 ± 2.40^a^

Values are presented as mean (SD). One-way analysis of variance was used to compare the white blood cells among the three groups. ^a^Compared with the group before chemotherapy, *P* < 0.05. ^b^Compared with the conventional group at the same time point, *P* < 0.05. ^c^Compared with the TCM herb group at the same time point, *P* < 0.05.

**Table 3:** Comparison of the changes in neutrophil count among the three groups (×10^9^/L, $\bar{x}$ *±* *s*).

Groups	1 d before	Day 5	Day 8	Day 11	Day 14	Day 21	Day 28
Conventional (*n* = 37)	5.41 ± 1.18	3.01 ± 1.41^a^	2.77 ± 1.17^a^	2.31 ± 1.24^a^	2.07 ± 1.14^a^	3.58 ± 1.23^a^	4.45 ± 1.34^a^
TCM herb (*n* = 35)	5.33 ± 1.38	3.88 ± 1.05^a,b^	3.48 ± 1.15^a^	3.03 ± 1.27^a^	2.63 ± 1.23^a^	4.10 ± 1.27^a^	4.83 ± 1.38
TEAS (*n* = 42)	5.22 ± 1.32	4.28 ± 1.54^a,b^	3.75 ± 1.21^a,b^	3.46 ± 1.31^a,b^	3.18 ± 1.29^a,b^	4.67 ± 1.31^b^	5.06 ± 1.44

Values are presented as mean (SD). One-way analysis of variance was used to compare the neutrophil count among the three groups. ^a^Compared with the group before chemotherapy, *P* < 0.05. ^b^Compared with the conventional group at the same time point, *P* < 0.05. ^c^Compared with the TCM herb group at the same time point, *P* < 0.05.

**Table 4:** Comparison of the changes in platelet count among the three groups (×10^9^/L, $\bar{x}$ *±* *s*).

Groups	1 d before	Day 5	Day 8	Day 11	Day 14	Day 21	Day 28
Conventional (*n* = 37)	258.3 ± 79.0	201.0 ± 55.7^a^	188.2 ± 65.8^a^	181.3 ± 84.3^a^	192.8 ± 95.8^a^	239.3 ± 70.4	247.6 ± 84.8
TCM herb (*n* = 35)	264.7 ± 88.4	216.3 ± 57.9^a^	213.7 ± 70.3^a^	192.1 ± 74.7^a^	201.9 ± 89.3^a^	213.9 ± 82.6^a^	221.5 ± 80.2^a^
TEAS (*n* = 42)	271.6 ± 98.4	264.7 ± 64.1^b,c^	251.3 ± 74.9^b,c^	236.7 ± 74.9^b,c^	238.3 ± 75.9^b^	254.8 ± 81.8^c^	244.1 ± 88.8

Values are presented as mean (SD). One-way analysis of variance was used to compare the platelets count among the three groups. ^a^Compared with the group before chemotherapy, *P* < 0.05. ^b^Compared with the conventional group at the same time point, *P* < 0.05. ^c^Compared with the TCM herb group at the same time point, *P* < 0.05.

**Table 5:** Comparison of the changes in hemoglobin level among the three groups (g/L, $\bar{x}$ *±* *s*).

Groups	1 d before	Day 5	Day 8	Day 11	Day 14	Day 21	Day 28
Conventional (*n* = 37)	130.58 ± 13.55	125.45 ± 15.38	122.46 ± 17.01	121.89 ± 15.33	122.09 ± 12.63	124.03 ± 12.50	121.80 ± 14.15
TCM herb (*n* = 35)	129.47 ± 15.97	128.41 ± 14.64	125.92 ± 15.91	126.58 ± 16.53	135.03 ± 28.06^b^	125.77 ± 11.78	124.71 ± 22.65
TEAS (*n* = 42)	121.11 ± 14.37	126.13 ± 12.38	120.58 ± 14.15	124.68 ± 14.23	126.94 ± 15.89	125.35 ± 15.43	120.75 ± 16.00

Values are presented as mean (SD). One-way analysis of variance was used to compare the hemoglobin level among the three groups. ^a^Compared with the group before chemotherapy, *P* < 0.05. ^b^Compared with the conventional group at the same time point, *P* < 0.05. ^c^Compared with the TCM herb group at the same time point, *P* < 0.05.

**Table 6:** Comparison of comfort scores among the three groups (score).

Groups	1 d before	Day 5	Day 11	Day 21
Conventional (*n* = 37)	82.85 ± 9.73	70.18 ± 9.34^a^	72.11 ± 9.47^a^	81.85 ± 9.50
TCM herb (*n* = 35)	80.05 ± 9.97	74.08 ± 9.67	76.58 ± 10.23	82.47 ± 9.78
TEAS (*n* = 42)	79.41 ± 9.56	78.31 ± 10.21^b^	80.07 ± 10.44^b^	82.55 ± 9.43

Values are presented as mean (SD). One-way analysis of variance was used to compare the comfortable score among the three groups. ^a^Compared with the group before chemotherapy, *P* < 0.05. ^b^Compared with the conventional group at the same time point, *P* < 0.05. ^c^Compared with the TCM herb group at the same time point, *P* < 0.05.

## Data Availability

The data used to support the findings of this study are available from the corresponding author upon request.

## References

[1] Yoshida Y., Watanabe S. (2018). Primary lung cancer surgery-clinical trial results. *Cancer & Chemotherapy*.

[2] Siegel R. L., Miller K. D., Jemal A. (2019). Cancer statistics, 2019. *CA: A Cancer Journal for Clinicians*.

[3] Nagasaka M., Gadgeel S. M. (2018). Role of chemotherapy and targeted therapy in early-stage non-small cell lung cancer. *Expert Review of Anticancer Therapy*.

[4] Gao X. Y., Zhou C. C., Gu F. (2018). Prospective study of risk factors for myelosuppression after first chemotherapy in patients with non-small cell lung cancer. *Journal of Tongji University (Medical Edition)*.

[5] Zhao Z. F., He X. R., Zhang Q. (2017). Meta-analysis of diyushengbai tablets in the treatment of leukopenia induced by tumor chemotherapy. *NorthWest Pharmaceutical*.

[6] Zhao G. R., Wang Y. D., Chen Y. D. (2005). The effects of diyushengbai table on leucocyte decreasing in chemotherapy of non-small cell lung cancer. *Cancer Research and Treatment*.

[7] Peng Y. Y., Tian N. (2019). Discussion on the idea of acupuncture and moxibustion for preventing and treating tumor bone marrow suppression by meridian points. *Massage techniques in rehabilitation medicine*.

[8] Liu M., Shen W. D., Cheng S. D. (2018). Effect of acupuncture on bone marrow suppression and quality of life in patients with colorectal cancer chemotherapy. *Journal of Traditional Chinese Medicine*.

[9] Lin W. B., Zhou J. Y., Jiang B. (2017). Effect of ginger moxibustion on myelosuppression caused by chemotherapy of multiple myeloma. *Shanghai Acupuncture Journal*.

[10] Miao Y. D., Li Y. H., Shen H. Y. (2019). Advances in research on traditional Chinese medicine for myelosuppression induced by chemotherapy in malignant tumors. *Journal of Traditional Chinese Medicine*.

[11] Zhao F. C., Wang Z. Y., Ye C. Y. (2020). Effect of transcutaneous electrical acupoint stimulation on one-lung ventilation-induced lung injury in patients undergoing esophageal cancer operation. *Evidence-Based Complementary and Alternative Medicine*.

[12] Zeng C., Wang S. H., Wang G. F. (2014). Development and application of behavioral anchor rating scale for clinical nursing teachers. *Qilu Nursing Journal*.

[13] Cao S., Wang S., Ma H. (2016). Genome-wide association study of myelosuppression in non-small-cell lung cancer patients with platinum-based chemotherapy. *The Pharmacogenomics Journal*.

[14] Jiang N., Chen X.-C., Zhao Y. (2013). Analysis of the risk factors for myelosuppression after concurrent chemoradiotherapy for patients with advanced non-small cell lung cancer. *Supportive Care in Cancer*.

[15] Yu C. F., Jiang Z. H., Hou A. H. (2017). Shen-cao granules formulated based on traditional Chinese medicine alleviates bone marrow suppression caused by platinum-based anticancer reagents. *Medicine (Baltimore)*.

[16] Fu H., Chen B., Hong S., Guo Y. (2015). Acupuncture Therapy for the treatment of myelosuppression after chemotherapy: a literature review over the past 10 Years. *Journal of Acupuncture and Meridian Studies*.

[17] Gao L., Luo J. (2019). Effect of percutaneous acupoint electrical stimulation on bone marrow suppression and adverse emotions in patients with lung cancer chemotherapy. *Zhong Guo Zhong Yi Yao Xian Dai Yuan Cheng Jiao Yu*.

[18] Xu H. D., Jia Y. J., Chen J. (2014). Current status and meridian analysis of moxibustion-induced myelosuppression induced by chemotherapy. *Cancer*.

[19] Xiao X. H., Jia R. M., Li H. K. (2019). Therapeutic effect of moxibustion at Zusanli acupoint on bone marrow suppression after chemotherapy in patients with extensive small cell lung cancer. *Chinese Medical Journal*.

[20] Hou L., Gu F., Gao G., Zhou C. (2017). Transcutaneous electrical acupoint stimulation (TEAS) ameliorates chemotherapy-induced bone marrow suppression in lung cancer patients. *Journal of Thoracic Disease*.

[21] Hou L. L., Gu F., Zhou C. C. (2016). Effect of percutaneous acupoint electrical stimulation on prevention of bone marrow suppression after chemotherapy in patients with lung cancer. *Chinese Journal of Nursing*.

[22] Huo Y. P. (2019). Study on the effect of comfortable nursing on treatment compliance and quality of life in patients with lung cancer undergoing radiotherapy and chemotherapy. *Chinese Medicine Guide*.

[23] Bilgiç Ş, Acaroğlu R. (2017). Effects of listening to music on the comfort of chemotherapy patients. *Western Journal of Nursing Research*.

[24] He X. J., Xu X. X., Hu R. Z. (2018). Effect of acupoint massage combined with acupoint application on quality of life in patients with primary lung cancer during chemotherapy. *Zhong Xi Yi Jie He Hu Li*.

[25] Shao Y. (2016). Clinical evaluation of percutaneous acupoint electrical stimulation during chemotherapy in patients with non-small cell lung cancer. *Zhong Xi Yi Jie He Hu Li*.

